# A Novel Classification and Identification Scheme of Emitter Signals Based on Ward's Clustering and Probabilistic Neural Networks with Correlation Analysis

**DOI:** 10.1155/2018/1458962

**Published:** 2018-11-05

**Authors:** Xiaofeng Liao, Bo Li, Bo Yang

**Affiliations:** ^1^College of Electronic and Information Engineering, Southwest University, 400715 Chongqing, China; ^2^Department of Mathematics, Army Logisticals University of PLA, 401331 Chongqing, China

## Abstract

The rapid development of modern communication technology makes the identification of emitter signals more complicated. Based on Ward's clustering and probabilistic neural networks method with correlation analysis, an ensemble identification algorithm for mixed emitter signals is proposed in this paper. The algorithm mainly consists of two parts, one is the classification of signals and the other is the identification of signals. First, self-adaptive filtering and Fourier transform are used to obtain the frequency spectrum of the signals. Then, the Ward clustering method and some clustering validity indexes are used to determine the range of the optimal number of clusters. In order to narrow this scope and find the optimal number of classifications, a sufficient number of samples are selected in the vicinity of each class center to train probabilistic neural networks, which correspond to different number of classifications. Then, the classifier of the optimal probabilistic neural network is obtained by calculating the maximum value of classification validity index. Finally, the identification accuracy of the classifier is improved effectively by using the method of Bivariable correlation analysis. Simulation results also illustrate that the proposed algorithms can accurately identify the pulse emitter signals.

## 1. Introduction

Under the conditions of the rapid development of modern information technology, various types of communication equipment, such as radar and radio navigation equipment, radio and television equipment, and electronic computer and peripheral equipment, have been used by military and large technology companies. Its applications have been extended from the ground, air, and sea to outer space. In order to obtain important information promptly, accurately, and effectively, the signal characteristics derived from the same types of communication sources need to be extracted and analyzed. The realization of identification from general communication signals to individual signals has a significance to win the initiative. Therefore, many researchers have devoted to the study of the identification of emitter signals. Their purpose is to further improve the accuracy of signal classification and identification.

The earliest research on the identification of emitter signals began in the 1970s, and it was one of the key technologies in Electronic Warfare systems. At present, the identification of emitter is mainly based on two modeling techniques: syntactic pattern-based methods and parametric pattern-based methods. In [1], Visnevski author had proposed a syntactic model to identify multifunction radar (MFR) signals. In the process of modeling, the MFRs were considered as stochastic discrete event systems that communicated information by use of radar word level modeling, radar phrase level modeling, and radar sentence level modeling. The radar word was a fixed arrangement of finite number of pulses, the radar phrase was a series of limited number of radar words, and the radar sentence was a combination of limited number of radar phrases. His simulation experiments had shown that the designed principle was effective for identifying MFRs. Based on the syntactic model of MFRs, Alex Wang and Vikram Krishnamurthy had used the stochastic context-free grammar to describe the behaviors of the MFR system, and some good results were obtained. Although the stochastic context-free grammar was a model for capturing the essential features of the MFR dynamics [2], it had some defects in estimating the parameters of stochastic context-free grammar. Therefore, the expectation maximization algorithm had been proposed by LP Dai et al. to estimate these parameters, which can be used to further estimate the characteristic parameters of MFR [3]. From this point of view, the ultimate goal of the modeling technique based on syntax was to find the feature parameters of emitters. It highlighted the importance of identification technology based on the parametric pattern. The feature parameter matching technique was a basic method of this pattern. The main characteristic of this method was to identify the emitter signals by matching the measured signal characteristic parameter vector with the corresponding characteristic parameters in the known database (the libraries of radar types). This method depended on the feature parameter database, and it can only be applied to emitter identification problem with invariant characteristic parameters. Moreover, the libraries of types had also an inherent uncertainty resulting inevitably from data collection methods. Therefore, Jan Matuszewski *et al.* had proposed the knowledge-based techniques to identify emitters [4, 5]. They think that the information of known radar platforms (knowledge), including position, intent, and recent operational history, plays an important role in the identification of emitters. Their approaches had been successfully used to identify some specific emitters. In order to fully utilize this knowledge to identify emitters, Janusz Dudczyk had proposed an idea of constructing Emitter DataBase based on the entity-relationship modeling. The entity-relationship diagram was introduced to realize this idea, which had pointed out a new direction for the construction of a complete and accurate Electronic Intelligence systems [6]. Meanwhile, artificial intelligence techniques and some optimized feature selection methods were used to improve the identification accuracy of emitter signals. In [7], the authors had proposed a vector neural network with a supervised learning algorithm which worked for signal classification and emitter identification. This network took carrier frequency, pulse width, and pulse repetition interval as inputs to complete the identification. In [8], the authors had proposed an identification method of radar signals based on immune radial-basis function neural network, which can improve the convergence speed and performance of the algorithm. In [9], a multichannel recognition system with an independent distance which was defined on impartiality condition had been proposed to identify the specific emitters. By modifying the distance in each special recognition channel, radio frequency, pulse width, and pulse repetition interval of radar signals can be extracted and classified into an appropriate class. Beyond that, some methods had been proposed to reduce the identification error rate. In [10], wavelet features were used as inputs of neural networks to identify emitters. In [11–13], the support vector machine was introduced to identify emitter signals. In [14, 15], the fuzzy c-means and probabilistic neural networks (PNN) were used to identify emitter signals. However, the determination of the optimal number of clusters was a major challenge for these methods, and they cannot effectively improve the identification accuracy. Therefore, Jawad *et al.* designed a clustering validity function in the hidden-layer output space of PNN to find the optimal number of clusters. Their methods were successfully applied to the classification of land use [16]. But, the method of determining the range of clustering number was subjective, which may reduce the identification accuracy of the algorithm. To overcome this deficiency and further improve accuracy, a classification and identification scheme of emitter signals based on the Ward clustering method (WCM) and PNN with correlation analysis was proposed in this paper. Its advantages are presented in three aspects:

1. The self-adaptive filtering, Ward's clustering, and clustering validity indexes are skillfully used to determine the scope of the optimal number of clusters.
2. The classification validity index *D* is flexibly used to find the optimal PNN classifier.
3. The probabilistic neural networks with Bivariable correlation analysis approach are proposed to improve the identification accuracy of emitter signals.

The rest of this paper is organized as follows. In Section 2, the classification and identification schemes including adaptive filtering, frequency spectrum, evaluation indexes, WCM and PNN classifier are introduced. The flowchart and the pseudocodes of the classification algorithms are designed in Section 3. The flowchart and the pseudocodes of the identification algorithm are given in Section 4. The identification experiments are also carried out in this section. The comparison discussions of different schemes are proposed in Section 5. Some innovations and applicable conditions of the proposed method are summarized in Section 6.

## 2. Classification Model of Emitter Signals

### 2.1. Self-Adaptive Filtering

In the field of engineering technology, the signal *x*(*n*) received at time *n* usually contains two parts. One is the useful signal *s*(*n*), which is what we need, and it enables us to understand the properties of the object to be studied. The other is the interference signal *x*_1_(*n*), which is what we do not need, and it prevents us from understanding the properties of the object to be studied. The actual signal will be obtained once the two parts are combined together. That is, *x*(*n*)=*s*(*n*)+*x*_1_(*n*).

**Figure 1:**
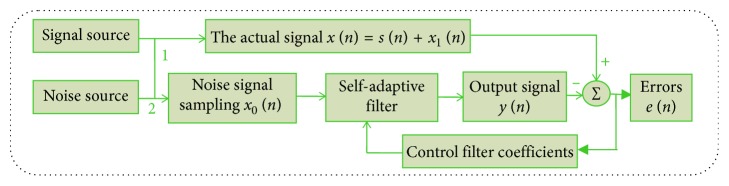
Flowchart of the self-adaptive filtering technology.

Weakening the interference signal *x*_1_(*n*) and maintaining or enhancing the useful signal *s*(*n*) are an important purpose of signal processing. The usual method is to use a frequency function *H*(*f*) to multiply the frequency spectrum *X*(*f*) of the signal *x*(*n*). This process is called filtering. Its essence is to weaken the interference signal and highlight the useful signal. Currently, the most widely used filters include Kalman filtering, Wiener filtering, median filtering, sequential statistical filtering, wavelet transform, self-adaptive filtering, etc. In terms of adaptability and filtering performance, one of the best filtering methods is the self-adaptive filtering, which is developed on the basis of Kalman filtering, Wiener filtering, and linear filtering. The most important feature of the self-adaptive filtering is that it can track the time-varying characteristics of input signals and eliminate the unknown interference contained in the signals. Self-adaptive filtering based on the least mean square algorithm is proposed by Widrow and Hoff, and it has been widely used in many fields because of its simplicity, robustness, and easy implementation. The principle diagram of self-adaptive filtering technology is shown in Figure 1.

Figure 1 presents the schematic diagram of noise elimination for self-adaptive filter. The actual signal *x*(*n*) contains interference signal *x*_1_(*n*) generated from signal channel 1. In order to eliminate it, the noise signal *x*_0_(*n*) which is independent of *s*(*n*) but related to *x*_1_(*n*) must be sampled from noise source through the signal channel 2. The main function of the self-adaptive filter is to process *x*_0_(*n*) so that the output *y*(*n*) approximates to *x*_1_(*n*). Under the condition of convergence of filtering algorithm, the output *e*(*n*) of the system approximates to *s*(*n*) when *y*(*n*) approaches *x*_1_(*n*). The iterative formulas of self-adaptive filtering algorithm based on the least mean square are defined as follows [17]:

(1) $$\begin{array}{c} e\left(n\right)=d\left(n\right)-\omega^{T}\left(n\right)X\left(n\right),\\ \omega \left(n+1\right)=\omega \left(n\right)+2\mu e\left(n\right)X\left(n\right), \end{array}$$

where *d*(*n*) is the desired signal; *X*(*n*)=[*x*(*n*), *x*(*n* − 1), ⋯ ,*x*(*n* − *M*+1)]^*T*^ is the input signal vector at time *n*; *M* is the length of filter; and *μ* is the fixed step size and satisfies 0 < *μ* < 1/*MP*_*in*_, where *P*_*in*_ is the input power of filter. *ω*(*n*)=[*w*_0_(*n*), *w*_1_(*n*), ⋯ ,*w*_*M*−1_(*n*)]^*T*^ is the weight vector of *M* order adaptive filter at time *n*.*ω*(*n*)=0 at initial time. *y*(*n*)=*ω*^*T*^(*n*)*X*(*n*) represents the actual output signal of filter. In noise elimination applications, *x*(*n*) is usually used as the desired signal *d*(*n*) and *x*_0_(*n*) is used as the input signal of the filter to eliminate *x*_1_(*n*). After many iterations, the difference between *d*(*n*) and *y*(*n*) is the estimate of signal *s*(*n*). This algorithm has the advantages of small amount of computation, easy implementation, and stable performance, but its convergence speed is relatively slow. Therefore, the authors proposed a variable step adaptive filtering algorithm based on bell-shaped function [18]. The variable step size is given as follows:

(2) $$\begin{array}{c} \mu \left(n\right)=\mu_{\max}\left(1-e^{-a{\left|e\left(n\right)\right|}^{b}}\right), \end{array}$$

where *μ*_max_ ∈ (0,1/*MP*_*in*_) is the maximum step size that can maintain the convergence of the adaptive filtering algorithm. Experiments show that this algorithm can effectively improve the convergence speed and reduce the steady-state error when *a*=0.08 and *b*=4. In subsection 3, the method is used to eliminate the interference signal of emitters.

### 2.2. The Spectrum of Signals

In order to classify and identify signals, it is necessary to analyze the frequency spectrum and energy spectrum of the signals. From the electrical knowledge, *P*=*V*^2^/*R*, where *V* represents voltage and *R* represents resistance. If the resistance *R*=1 and *V* is replaced by signal *x*(*t*), the instantaneous energy is *x*^2^(*t*). Thus, the total energy of the signals can be expressed as ∫_−*∞*_^+*∞*^*x*^2^(*t*) *dt*. According to Parseval's theorem, it can be obtained by the following equation:

(3) $$\begin{array}{c} \int_{-\infty }^{+\infty }{\left|x\left(t\right)\right|}^{2\;}dt=\int_{-\infty }^{+\infty }{\left|X\left(f\right)\right|}^{2\;}df, \end{array}$$

where

(4) $$\begin{array}{c} X\left(f\right)=\int_{-\infty }^{+\infty }x\left(t\right)e^{-2\pi ift}\;dt, \end{array}$$

is the Fourier transform for signal *x*(*t*) and *f* represents the frequency of signal *x*(*t*). |*X*(*f*)| is called the amplitude spectrum and arg*X*(*f*) is called the phase spectrum of *x*(*t*). The frequency spectrum of *x*(*t*) consists of amplitude spectrum and phase spectrum. |*X*(*f*)|^2^ is called energy spectrum density. Equation (3) indicates that the energy of *x*(*t*) is closely related to |*X*(*f*)|^2^, and it can be obtained by calculating its integral on (−*∞*, +*∞*). Therefore, we can obtain the frequency distribution and energy distribution of each signal by analyzing the spectrum of the signal. Then, the key amplitude and the frequency of energy distribution can be further obtained, which provide conditions for directly observing the similarities and differences of paired signals.

### 2.3. The Ward Clustering Method

As a hierarchical agglomerate cluster algorithm, the WCM has a wide range of applications [19–21]. First, it is started by accepting each node as a separate cluster. Then, the clusters with minimum distance between themselves are combined in pairs at each stage of the algorithm. This smallest distance is called the Ward distance and defined as follows:

(5) $$\begin{array}{c} d_{rs}=\frac{n_{r}\cdot n_{s}}{n_{r}+n_{s}}{\left\|{\bar{x}}_{r}-{\bar{x}}_{s}\right\|}^{2}, \end{array}$$

where *r* and *s* represent the two distinct clusters, *n*_*r*_ and *n*_*s*_ represent the number of data points of two clusters, respectively, ${\bar{x}}_{r}$ and ${\bar{x}}_{s}$ represent the center of the corresponding cluster, and ||·|| is Euclidean norm. The centers and cardinal numbers of the new cluster are updated according to the following equations:

(6) $$\begin{array}{c} {\bar{x}}_{r}^{\prime }=\frac{n_{r}\cdot {\bar{x}}_{r}+n_{s}\cdot {\bar{x}}_{s}}{n_{r}+n_{s}}, \end{array}$$

(7) $$\begin{array}{c} n_{r}^{\prime }=n_{r}+n_{s}. \end{array}$$

Ward's clustering algorithm has the following steps:

 
*Step 1.* Each sample point is treated as a cluster. At this time, the sum of squares of deviations for each cluster is equal to 0.
 
*Step 2.* Two arbitrary clusters are merged, and the sum of squares of deviations is calculated from Equations (5)–(7). If we assume that there are *N* clusters in total, it must be calculated *N*(*N* − 1)/2 times.
 
*Step 3.* The two clusters with the smallest squared sum of deviations are combined into one class. The method eventually aggregates all sample points into one class when the number of clusters is unknown.

If the number of clusters is known, the WCM can be directly used to classify the signal data after removing the noise. Otherwise, it can be obtained by analyzing the dendrogram of clustering. This is a rather subjective approach, which is difficult to help us finding the true number of clusters for a given data set. In recent research, the clustering validity indexes, such as Calinski-Harabasz (CH) index, Gap index, Silhouette (Silh) index, and Davies–Bouldin (DB) index, have been demonstrated to be the best validation tools for determining the optimal number of clusters [22–27].

#### 2.3.1. Calinski-Harabasz Index

For a given set *Y*={*y*_1_, *y*_2_, ⋯, *y*_*N*_}, assume that the dimension of each entity *y*_*i*_ is *v*, *i*=1,2, ⋯, *N*. *K* nonempty disjoint cluster sets *S*={*S*_1_, *S*_2_, ⋯, *S*_*K*_} around the centroid set *C*={*c*_1_, *c*_2_, ⋯, *c*_*K*_} can be obtained by minimizing the within-cluster distance *W*_*K*_:

(8) $$\begin{array}{c} W_{K}=\overset{K}{\underset{k=1}{\sum }}\sum_{y_{i}\in S_{k}}d\left(y_{i},c_{k}\right), \end{array}$$

where *d*(*y*_*i*_, *c*_*k*_),  *k*=1,2, ⋯, *K* represents the squared Euclidean distance between the entity *y*_*i*_ and the centroid *c*_*k*_, that is,

(9) $$\begin{array}{c} d\left(y_{i},c_{k}\right)=\sum_{j\in v}{\left(y_{ij}-c_{kj}\right)}^{2}. \end{array}$$

Then, the CH index is defined as follows [24]:

(10) $$\begin{array}{c} \text{CH}\left(K\right)=\frac{\left(T_{1}-W_{K}\right)/\left(K-1\right)}{W_{K}/\left(N-K\right)}, \end{array}$$

where *W*_*K*_ is defined as in (8), and *T*_1_ can be calculated by $T_{1}={\sum_{i=1}^{N}\sum_{j\in v}\left(y_{ij}-{\bar{y}}_{j}\right)}^{2}$.

The CH index can reflect the compactness of the cluster by means of the overall within-cluster variance. The separation degree of the clusters can be reflected by the overall between-cluster variance. Therefore, a good clustering scheme corresponds to a higher value of CH index.

#### 2.3.2. Silhouette Index

For each entity *y*_*i*_, its silhouette value measures the similarity between the entity *y*_*i*_ and the points in its own cluster, when compared to the points in other clusters. This similarity is reflected by measuring the distance between the entity *y*_*i*_ and the points derived from different clusters. The silhouette value of the entity *y*_*i*_ is defined as follows [24]:

(11) $$\begin{array}{c} s\left(y_{i}\right)=\frac{b\left(y_{i}\right)-a\left(y_{i}\right)}{\max\left\{a\left(y_{i}\right),b\left(y_{i}\right)\right\}}, \end{array}$$

where *a*(*y*_*i*_) is the average distance from the entity *y*_*i*_ ∈ *S*_*k*_ to all other points *y*_*j*_ ∈ *S*_*k*_, *b*(*y*_*i*_) is the minimum distance from the entity *y*_*i*_ to all other points *y*_*j*_, which satisfies *y*_*j*_ ∈ *S*_*l*_, *y*_*i*_ ∉ *S*_*l*_, *l* ≠ *k*. Therefore, −1 ≤ *s*(*y*_*i*_) ≤ 1. If *s*(*y*_*i*_) is close to zero, the entity *y*_*i*_ could be assigned to another cluster. A negative value of *s*(*y*_*i*_) indicates that the corresponding assignment seriously damages cluster cohesion, and the clustering result of *y*_*i*_ is not advisable. *y*_*i*_ is well matched to its own cluster when *s*(*y*_*i*_) is close to 1. Finally, the validity of the whole clustering can be quantified by Silh index, and it is defined as follows:

(12) $$\begin{array}{c} \text{Silh}\left(K\right)=\overset{N}{\underset{i=1}{\sum }}\frac{s\left(y_{i}\right)}{N}. \end{array}$$

The Silh index can be used with any distance metric, including the Manhattan distances and Euclidean distances.

#### 2.3.3. Davies–Bouldin Index

A good partition should have a larger intercluster separation degree and stronger within-cluster homogeneity and compactness. The DB index is proposed based on this idea [26]. More concretely, it is constructed by a ratio of within-cluster and between-cluster distances. The DB index is defined as follows:

(13) $$\begin{array}{c} \text{DB}\left(K\right)=\left(\overset{K}{\underset{k=1}{\sum }}\max_{k\neq j}\left\{\frac{\left({\bar{d}}_{k}+{\bar{d}}_{j}\right)}{d_{kj}}\right\}\right)/K, \end{array}$$

where

(14) $$\begin{array}{c} {\bar{d}}_{k}={\left(\frac{1}{\left|S_{k}\right|}\overset{\left|S_{k}\right|}{\underset{i=1}{\sum }}{\left|y_{i}-c_{k}\right|}^{q}\right)}^{1/q}, \end{array}$$

represents the average distance between each point *y*_*i*_ in cluster *k* and the centroid of cluster*k*. |*S*_*k*_| is the number of points in cluster *k*. If *q*=1, ${\bar{d}}_{k}$ is the average Euclidean distance between the points in cluster *k* to the centroid of cluster *k*. If *q*=2, ${\bar{d}}_{k}$ is the standard deviation of the distance of points in cluster *k* to the center of cluster *k*. When *k* in ${\bar{d}}_{k}$ is replaced by *j*, ${\bar{d}}_{j}$ can be obtained. In addition, *d*_*kj*_ can be calculated according to the following equation:

(15) $$\begin{array}{c} d_{kj}={\left(\overset{v}{\underset{h=1}{\sum }}{\left|{\bar{c}}_{kh}-{\bar{c}}_{jh}\right|}^{p}\right)}^{1/p}. \end{array}$$

It represents the distances between the centroids of the *k*th and the *j*th clusters. ${\bar{c}}_{kh}$ is the *h*th component of the centroid of cluster *k* and *d*_*kj*_ is the Minkowski metric of the centroids which characterizes clusters *k* and *j*. Specifically, *p*=1, *d*_*kj*_ is the Manhattan distance between centroids *p*=2, *d*_*kj*_ is the Euclidean distance between centroids.

The DB index can reflect the degree of within-cluster dispersion and between-cluster separation. So, the true number of clusters may be determined according to the minimum value of the DB index.

#### 2.3.4. Gap Index

Robert Tibshirani et al. proposed the gap statistic method for estimating the number of clusters in a set of data [27]. A graph of the within-cluster dispersion versus the number of clusters *k* for a clustering procedure shows that the within-cluster dispersion decreases monotonically as *k* increases, but from some *k* onwards, the decrease becomes flatter obviously. Such position is called ‘elbow', it often implies the appropriate number of clusters. The gap criterion gives an approach to estimate the number of clusters by locating this ‘elbow'. Therefore, under this criterion, the optimal number of clusters occurs at the largest gap value. The Gap index is defined as follows:

(16) $$\begin{array}{c} {\text{Gap}}_{N}\left(K\right)=E_{N}\left\{\log\left(U_{K}\right)\right\}-\log\left(U_{K}\right), \end{array}$$

where *N* represents the number of points, *K* represents the number of clusters that are evaluated, *U*_*K*_ defined in (17) represents the within-cluster dispersion degree.

(17) $$\begin{array}{c} U_{K}=\overset{K}{\underset{k=1}{\sum }}\frac{1}{2N_{k}}H_{k}, \end{array}$$

where *N*_*k*_ is the number of points in cluster *k*, *H*_*k*_ is the sum of the distances of any two points in the *k*th cluster. The expected value *E*_*N*_{*log*(*U*_*K*_)} is determined by Monte Carlo sampling from a reference distribution. The Gap index can also be used for any distance metric.

The WCM belongs to unsupervised categorization technique, which can help us find the centroid of each cluster. However, the classification accuracy of this method is limited, which makes the method not able to be used directly for signal recognition. Comparatively, PNN can effectively improve the accuracy of classification and identification [28, 29].

### 2.4. Probabilistic Neural Network Classifier

**Figure 2:**
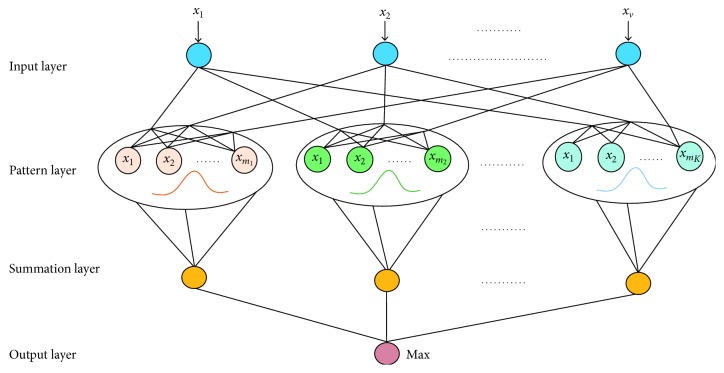
Flowchart of the PNN.

As a method of nonparametric Parzen windows estimation, PNN is first proposed by Specht. It is a nonlinear classification technique and essentially a parallel algorithm based on Bayesian minimum risk criterion [30]. Given a sample to be identified *x*, its posterior probability *P*(*S*_*k*_|*x*) can be obtained by PNN classifier. However, if the probability densities of the classes to be separated are unknown, the training samples with known identity need to be used to estimate them. Finally, the trained PNN is used to determine the identity of *x*. A typical PNN classifier consists of an input layer, a pattern layer (hidden layer), a summation layer and a output layer. The flowchart of the PNN is shown in Figure 2.

The input layer neurons are used to receive values from training samples and send data to the neurons in the pattern layer, which is fully connected to the input layer. The number of neurons in the input layer is equal to the length of the input vector. The number of neurons in the pattern layer is the same as the number of training samples. Here, all neurons are collected into different groups, and the *i*th neuron in group *k* corresponds to a Gaussian function *f*_*i*_^(*k*)^(*x*, *σ*), *i*=1,2, ⋯, *m*_*k*_, where *m*_*k*_ represents the number of neurons in group *k*, *k*=1,2, ⋯, *K*. Gaussian function which is also called probability density function is defined as follows:

(18) $$\begin{array}{c} f_{i}^{\left(k\right)}\left(x,\sigma \right)=\frac{1}{{\left(2\pi \right)}^{v/2}\sigma^{v}}\exp\left[-\overset{v}{\underset{j=1}{\sum }}\frac{{\left(x_{ij}^{\left(k\right)}-x_{j}\right)}^{2}}{2\sigma^{2}}\right], \end{array}$$

where *v* is the dimension of the input vector *x*=(*x*_1_,  *x*_2_, ⋯ , *x*_*v*_), *x*_*j*_ is the *j*th component of the input vector *x*, *x*_*ij*_^(*k*)^ is the *j*th component of the *i*th neuron in class *k*. The so-called smoothing parameter *σ* ∈ (0,1) determined experimentally by comparing their corresponding classification accuracy plays an important role in estimation error of the PNN classifier. The outputs of pattern layer are connected to the summation units depending on the class of patterns. There is one neuron for each group, and each neuron in summation layer sums the outputs derived from the pattern layer neurons as follows:

(19) $$\begin{array}{c} p_{k}\left(x\right)=\frac{1}{{\left(2\pi \right)}^{v/2}\sigma^{v}m_{k}}\overset{m_{k}}{\underset{i=1}{\sum }}\exp\left[-\overset{v}{\underset{j=1}{\sum }}\frac{{\left(x_{ij}^{\left(k\right)}-x_{j}\right)}^{2}}{2\sigma^{2}}\right]. \end{array}$$

Finally, the output layer neuron output a number 1 and multiple numbers 0. The value of 1 corresponds to the classifier's decision result for input vectors. More specifically, the input vector *x* belongs to class *k* if *p*_*k*_(*x*) > *p*_*k*′_(*x*) for all *k*′=1,2, ⋯, *K* and *k* ≠ *k*′.

Hence, the main purpose of training PNN is to find the optimal estimate of probability density function according to the training samples and their labels, to ensure that the classifier works at the condition of minimum error rate and risk. When the samples to be identified are sent to the pattern layer, the output of each neuron is calculated according to the trained density function. Finally, the identified results are obtained through computations in the summation layer and output layer. Due to the following advantages, it is a wise choice to use PNN as a further classifier to classify signals [31]:

1. It has a simple structure, and it is easy to train. In the PNN based on probability density function estimation, the weight of the neuron in pattern is directly taken from the input sample value.
2. The training process of the network is simple, and there is no need to retrain for a long time when adding or reducing the number of groups.
3. It is not easy to produce local optimal solution, and its precision is higher than that of other classification approaches. No matter how complex the classification problem is, as long as there are enough training samples, the optimal solution under the Bayes criterion can be obtained.

## 3. Classification Scheme and Experiments of Emitter Signals

### 3.1. Flowchart and Algorithms of Classification Scheme

**Figure 3:**
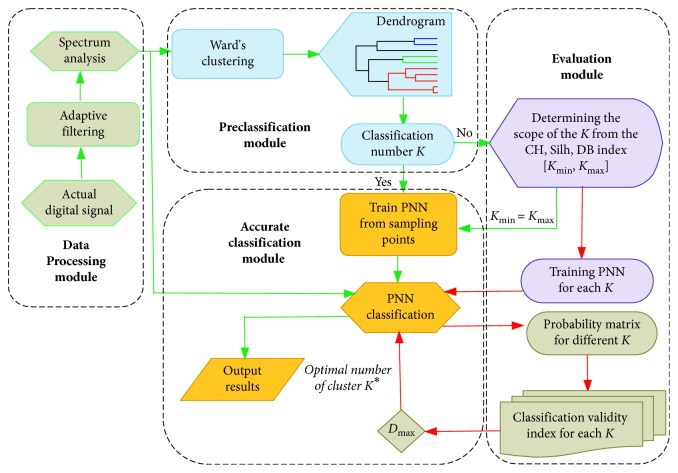
Flowchart of signal classification.

The flowchart of the proposed classification algorithms is shown in Figure 3.

Figure 3 indicates that the proposed scheme is composed of four modules, that is, data processing module, preclassification module, evaluation module, and accurate classification module. In the evaluation module, the clustering validity indexes are used to determine the range of *K* if it is unknown. For each *K* ∈ [*K*_Min_, *K*_Max_], the classification validity index *D* is calculated as follows [16]:

(20) $$\begin{array}{c} D\left(K\right)=\frac{K\sum_{j=1}^{N_{1}}\max_{1\leq k\leq K}\left\{p_{kj}\right\}-N_{1}}{N_{1}\left(K-1\right)}, \end{array}$$

where *N*_1_ is the number of input vectors, *p*_*kj*_ is the element of the matrix *Q* of size *K* × *N*_1_ in the output of PNN's pattern layer representing the membership of the *j*th input vector to the cluster *k*. When *N*_1_=1, *p*_*k*1_ is equal to *p*_*k*_(*x*) presented in (19). When *N*_1_ > 1, matrix *Q*=(*p*_*kj*_)_*K*×*N*_1__ can be obtained by PNN. max_1≤*k*≤*K*_{*p*_*kj*_} is the largest element of the *j*th column in the matrix *Q*. Equation (20) indicates that *D*(*K*) is a nonlinear function related to *K*, when *N*_1_ ≠ ∑_*j*=1_^*N*_1_^max_1≤*k*≤*K*_{*p*_*kj*_}. Therefore, *K* corresponding to the maximum value of *D*(*K*) is the optimal number of clusters *K*^*∗*^.

The pseudocodes are listed in Algorithm 1 if *K* is known.

If the classification number *K* is unknown, WCM and clustering validity indexes are used to determine the range of *K*. The corresponding pseudocodes are listed in Algorithm 2.

The algorithms show that the supervised learning PNN is used to classify samples. Therefore, the training (teaching) samples must be selected first. By Ward clustering method, we have obtained a preliminary classification of all samples. That is, the identities of some samples have been determined, except for some boundary points, which need to be further determined by the trained PNN. Therefore, some labeled proximity points *x*_*kj*_(*k*=1,2, ⋯, *K*, *j*=1,2, ⋯, *J*_*k*_) around the center *c*_*k*_ can be selected to train PNN, where *J*_*k*_ represents the number of samples selected in class *k* and it should be preset, such as *J*_*k*_=⌈*a* · |*S*_*k*_|⌉, *a* ∈ [0.6, 0.8].

### 3.2. Classification Experiments

**Figure 4:**
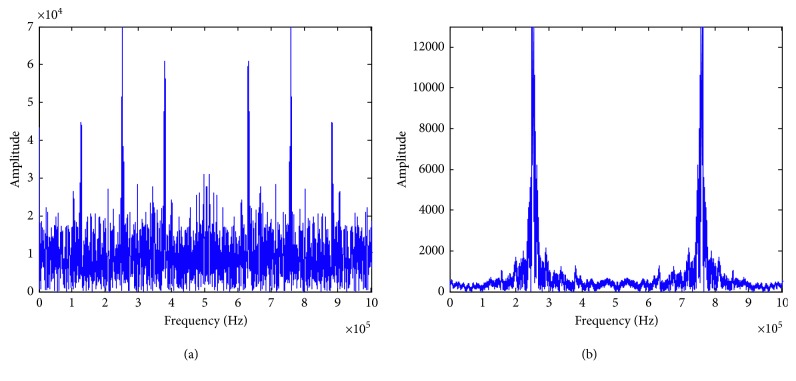
The double-sided amplitude spectrum of a sampling signal. (a) Before filtering; (b) after filtering.

A signal set *Rs* sampled from some pulse emitters is used to test the effectiveness of the proposed algorithms. Each emitter emits continuous signals in the pulse state. After a period of time, the receiver will receive multiple signals from all emitters. These signals are converted into digital signals by the analog-digital converter. The sampling frequency is 1.01 MHz. Signal samples *y*_*i*_(*i*=1,2, ⋯, 500) are randomly extracted from these digital signals. The signal set *Rs*=[*y*_1_, *y*_2_, ⋯,*y*_500_]^*T*^ and the dimension of *y*_*i*_ is 1024. Considering that each *y*_*i*_ is disturbed by signals from other emitters, the mean value of all signals is used as the noise signal *Is*=[*Is*(1)*Is*(2) ⋯ *Is*(1024)], where *Is*(*n*)=∑_*i*=1_^500^*y*_*i*_(*n*)/500, *n*=1,2, ⋯, 1024. First, the self-adaptive filtering is applied to process these signals. In this algorithm, the actual signal *x*(*n*) corresponds to *y*_*i*_(*n*) and the noise signal *x*_0_(*n*) corresponds to *Is*(*n*). Then, Fourier transform is used to obtain the amplitude spectrum of all processed signals. The amplitude spectrum of the thirteenth signal is shown in Figure 4.

**Figure 5:**
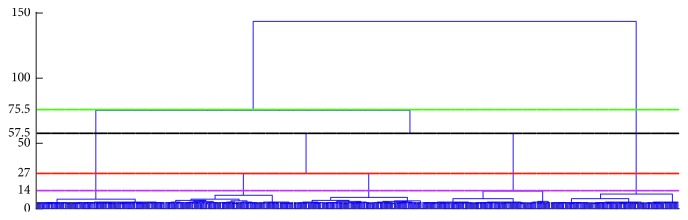
The dendrogram of the signals after using the WCM.

Figure 4(a) shows the sampling signal contains obvious white noise, which makes the feature of the useful signal unclear. However, most of the noises are removed after using the self-adaptive filtering. Moreover, the characteristics of the signals are highlighted so that the amplitude spectrum of them can be analyzed correctly. For these transformed signals, the clustering dendrogram can be obtained by using the WCM, and it is shown in Figure 5.

**Figure 6:**
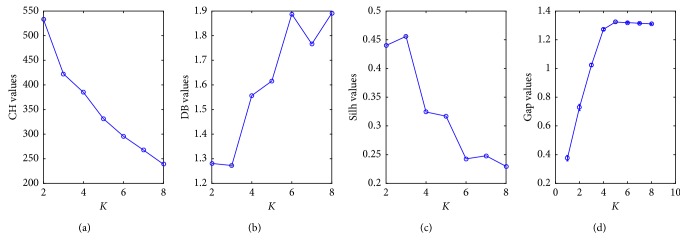
The number of clusters *K* versus the different evaluation indexes. (a) The number of clusters *K* versus CH values. (b) The number of clusters *K* versus DB values. (c) The number of clusters *K* versus Silh values. (d) The number of clusters *K* versus Gap values.

When the signals are divided into 3, 4, 5, and 6 classes, the intercluster distances are 75.5, 57.5, 27, and 14, respectively. The increments of the distance between the clusters are 13, 30.5, and 18 in turn. If the number of elements in each class is required relatively close, the ideal number of classifications is 3, 4, and 5. However, these numbers need to be further determined by the clustering validity indexes. The number of clusters *K* corresponding to different evaluation indexes is shown in Figure 6.

Figure 6 shows that the optimal number of clusters is 3 when the DB index and the Silh index are used, and it is 2 when the CH index is used. However, it becomes to 5 when the Gap index is used. Therefore, the optimal number of classifications should belong to the interval [2, 5]. In this case, the PNN needs to be used to obtain more accurate results.

**Figure 7:**
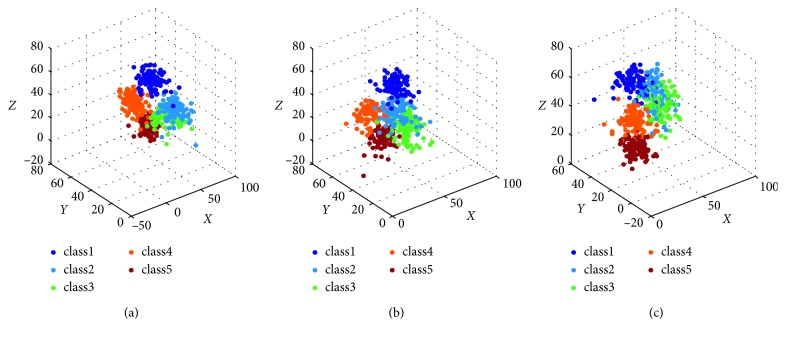
The scatter plots of classification results. (a) The classified results when *a*_1_=245, *a*_2_=562, *a*_3_=720. (b) The classified results when *a*_1_=268, *a*_2_=369, *a*_3_=547. (c) The classified results when *a*_1_=139, *a*_2_=367, *a*_3_=741.

For each *K* in this interval, seventy (*a*=0.7) sample points nearby each *c*_*k*_ are selected to train PNN classifiers. Then, the optimal classifier is obtained by calculating the maximum value of *D*(*K*). The results have shown that D(5)=1, while the other values are less than *D *(5). That is to say, the optimal number of classifications is 5. Since the size of matrix *Rs* is 500 × 1024, three columns are randomly selected as the *X*-axis, the *Y*-axis, and the *Z*-axis to plot the classification results diagram. Let

(21) $$\begin{array}{c} Ac_{k}=\left(\begin{array}{cccc} y_{1k}\left(1\right) & y_{1k}\left(2\right) & \cdots & y_{1k}\left(1024\right)\\ y_{2k}\left(1\right) & y_{2k}\left(2\right) & \cdots & y_{2k}\left(1024\right)\\ \vdots & \vdots & & \vdots \\ y_{M_{k}k}\left(1\right) & y_{M_{k}k}\left(2\right) & \cdots & y_{M_{k}k}\left(1024\right) \end{array}\right), \end{array}$$

represents a matrix consisting of all signals in class *k* after classifying, where *M*_*k*_ is the number of samples in class *k*, *y*_*jk*_ is the *j*th sample in class *k*, and *j*=1,2, ⋯, *M*_*k*_. Thus, *Ac*=[*Ac*_1_  *Ac*_2_ ⋯ *Ac*_5_]^*T*^ is a matrix of size 500 × 1024. If the data on columns *a*_1_, *a*_2_, *a*_3_ of *Ac* are selected to form a matrix $\bar{Ac}$ of size 500 × 3, each row of $\bar{Ac}$ is a three-dimensional array, that is, a point in the coordinate system. When *a*=0.5, *σ*=1, scatter plots of these data are shown in Figure 7. The first column of $\bar{Ac}$ corresponds to the *X*-axis, the second column of $\bar{Ac}$ corresponds to the *Y*-axis, and the third column corresponds to the *Z*-axis.

The experimental results show that all signal samples are divided into five classes, and each class contains 100 signals. Therefore, all signals can be thought to come from five emitters, and each emitter emits 100 signals. Although only three distribution figures of all classified signals are presented in Figure 7, in fact, in our experiments, we have obtained more than 1000 scatter plots which are drawn by randomly selecting three columns from matrix *Ac*. In these classification results, the distributions of sample sets are similar and the separations of them are obvious. Therefore, the proposed methods can effectively distinguish signals from different emitters.

## 4. Identification Scheme and Experiments of Emitter Signals

### 4.1. Flowchart and Algorithms of Identification Scheme

Besides classification, data identification is an important function of PNN. Since PNN is based on the maximum posterior probability, it will give the optimal solution under the Bayesian criterion, whether or not the samples to be identified *Ix*_*i*_ belong to the five determined classes that have been obtained in Section 3. So, if a sample belongs to one of them, it will accurately identify it. However, it may lose its function when the sample do not belong to them. At this time, the amplitude spectrum of all samples can be analyzed before, so that they can be identified whether they belong to the determined classes in advance. Considering that it is difficult to comparatively analyze the amplitude spectrum of the signals to be identified and each signal in every class, we adopt the curve fitting method to find the feature sequences of each class and the signals to be identified. Finally, the correlation degree of these sequences is calculated to get the preliminary identity information of the samples to be identified. This method is called Bivariable correlation analysis, and it is introduced as follows.

*Step 1*. *Simplifying amplitude spectrum.* Let *L*_*i*_ represent the length of the signal *Ix*_*i*_, *Fs*_*i*_ represent the Fourier transform of *Ix*_*i*_, |*Fs*_*i*_| is the amplitude spectrum of *Ix*_*i*_, |*Fs*_*i*_|=[*g*_*i*_(1)*g*_*i*_(2) ⋯ *g*_*i*_(*L*_*i*_)]. The sequence *F*_*i*_ of length *N*_2_ is extracted from |*Fs*_*i*_| which takes Δ*T*_*i*_ as the step size, where Δ*T*_*i*_=⌊*L*_*i*_/*N*_2_⌋. For each training sample *x*_*kl*_ in class *k*, the corresponding sequence *F*_*kl*_ can be obtained according to the same method, where *k*=1,2, ⋯, *K*^*∗*^, *l*=1,2, ⋯, *L*, *i*=1,2, ⋯, *p*, and *L* represents the number of training samples in class *k*, and *p* represents the number of samples to be identified.

*Step 2*. *Fitting curve.* The fitting curve ${\hat{z}}_{i}$ can be obtained by fitting the sequence *F*_*i*_ and the fitting curve ${\hat{y}}_{kl}$ in class *k* can be obtained by fitting the sequence *F*_*kl*_.

*Step 3*. *Constructing the feature sequence*. First, for different *k*, ${\hat{y}}_{kl}\left(t\right),t=1,2,\cdots ,T$ can be calculated after giving the upper bound *T*. Then, the *t*th signal feature of the class *k* can be obtained by the following equation:

(22) $$\begin{array}{c} {\hat{Y}}_{k}\left(t\right)=\frac{1}{L}\overset{L}{\underset{l=1}{\sum }}{\hat{y}}_{kl}\left(t\right). \end{array}$$

Finally, $Cs\left(k\right)=\left\{{\hat{Y}}_{k}\left(1\right),{\hat{Y}}_{k}\left(2\right),\cdots ,{\hat{Y}}_{k}\left(T\right)\right\}$ is the feature sequence of the class *k.* Similarly, the feature sequences $Z_{i}=\left\{{\hat{Z}}_{i}\left(1\right),{\hat{Z}}_{i}\left(2\right),\cdots ,{\hat{Z}}_{i}\left(T\right)\right\},\;\;\left(i=1,2,\cdots ,p\right)$ of the samples to be identified can be obtained.

**Figure 8:**
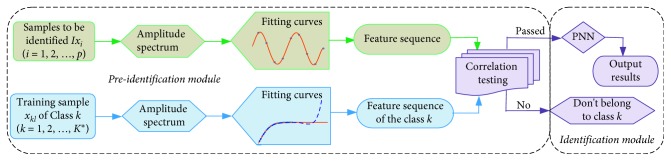
Flowchart of signal identification.

*Step 4*. *Correlation analysis.* The correlation coefficient between the *Z*_*i*_ and the *Cs*(*k*) can be calculated as the following equation:

(23) $$\begin{array}{c} r_{ik}=\frac{T\left(\sum_{t=1}^{T}{\hat{Y}}_{k}\left(t\right)\cdot {\hat{Z}}_{i}\left(t\right)\right)-\left(\sum_{t=1}^{T}{\hat{Y}}_{k}\left(t\right)\right)\cdot \left(\sum_{t=1}^{T}{\hat{Z}}_{i}\left(t\right)\right)}{\sqrt{\left[T\left(\sum_{t=1}^{T}{\hat{Y}}_{k}^{2}\left(t\right)\right)-{\left(\sum_{t=1}^{T}{\hat{Y}}_{k}\left(t\right)\right)}^{2}\right]\cdot \left[T\left(\sum_{t=1}^{T}{\hat{Z}}_{i}^{2}\left(t\right)\right)-{\left(\sum_{t=1}^{T}{\hat{Z}}_{i}\left(t\right)\right)}^{2}\right]}}, \end{array}$$

where $T\left(\sum_{t=1}^{T}{\hat{Y}}_{k}\left(t\right)\cdot {\hat{Z}}_{i}\left(t\right)\right)-\left(\sum_{t=1}^{T}{\hat{Y}}_{k}\left(t\right)\right)\cdot \left(\sum_{t=1}^{T}{\hat{Z}}_{i}\left(t\right)\right)$ represents the sample covariance of *Cs*(*k*) and *Z*_*i*_, T∑t=1TY^k2t−∑t=1TY^kt2 and T∑t=1TZ^i2t−∑t=1TZ^it2 are the sample standard variation of *Cs*(*k*) and *Z*_*i*_, respectively. The performance of the correlation test indicates that the sample to be identified belongs to the corresponding class when *r* ≥ 0.95. All samples that do not satisfy this condition should be removed. Finally, the remaining samples can be effectively identified by the trained PNN. The flowchart of the proposed identification scheme is shown in Figure 8.

Figure 8 shows that the flowchart mainly includes two blocks. One is the preidentification module, and the other is the identification module. The role of the previous module is to eliminate the sample with a small correlation to the determined classes. When the correlations between the samples to be identified and several different classes are high, PNN will accurately identify it based on Bayesian criteria. The pseudocodes are given in Algorithm 3, which is called identification algorithm.

Through the methods proposed in Section 3, all signal samples have been classified to five classes. That is to say, the identity of each sample has been determined. Thus, the identification algorithm consists of two parts. On the one hand, some proximity points *x*_*kl*_ around the center *c*_*k*_ of class *k* are selected to train PNN, *k*=1,2, ⋯, *K*^*∗*^, *l*=1,2, ⋯, *L*. *x*_*kl*_ is the input vector of the network, and its label is the output of the system. On the other hand, the trained PNN classifier is used to determine the identity of *Ix*_*i*_, *i*=1,2, ⋯, *p*.

If the dimension of *Ix*_*i*_ is greater than *x*_*kl*_, the method of adding time window can be used to adjust their dimensions. Let *D*_*x*_ represent the dimension of *x*_*kl*_ and *D*_*Ix*_ represent the dimension of *Ix*_*i*_. The time window *Ts*=⌊*D*_*Ix*_/*D*_*x*_⌋ is used to reduce the value of *D*_*Ix*_. Thus, the adjusted samples to be identified $\bar{I}x_{i}=\left[Ix_{i}\left(\text{1}\right)\text{,}\;\;Ix_{i}\left(\text{1}\right)+Ts\text{,}\;\;\cdots \text{,}\;\;Ix_{i}\left(\text{1}\right)+\left(D_{x}-1\right)Ts\right]$. When *D*_*Ix*_ < *D*_*x*_, the same method can be used to reduce the dimension of *x*_*kl*_ and make it consistent with *D*_*Ix*_. At this moment, *D*_*Ix*_ and *D*_*x*_ need to be exchanged.

### 4.2. Identification Experiments

**Figure 9:**
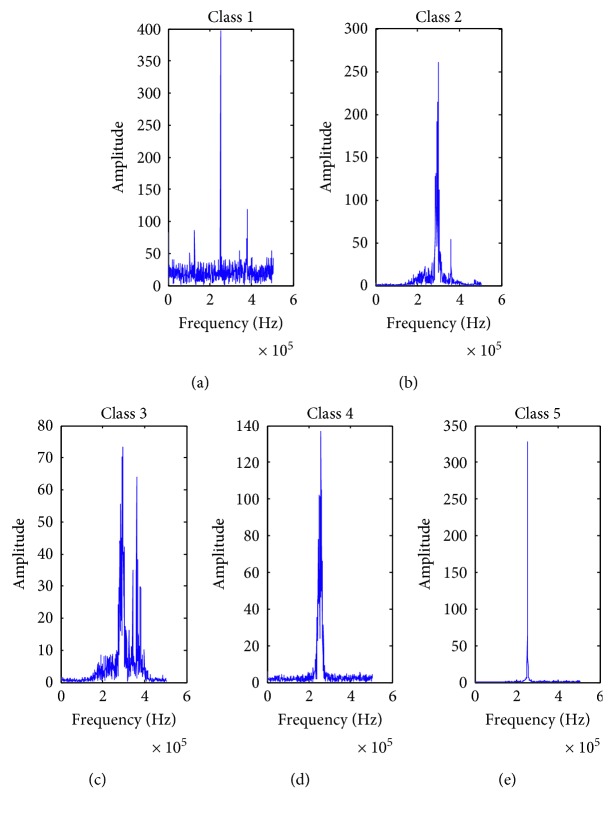
The single-sided amplitude spectrum of class *k*, *k*=1,2, ⋯, 5. (a) *k*=1; (b) *k*=2; (c) *k*=3; (d) *k*=4; (e) *k*=5.

**Figure 10:**
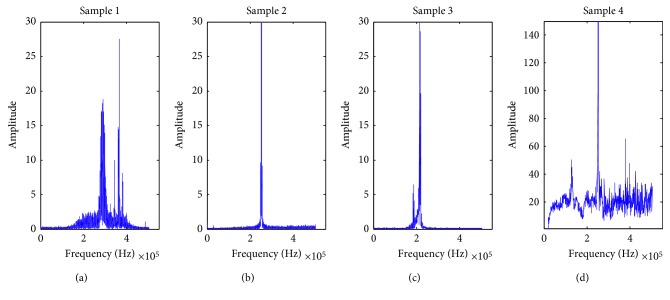
The double-sided amplitude spectrum of $\bar{I}x_{i}\left(i=1,2,3,4\right)$. (a) *i*=1; (b) *i*=2; (c) *i*=3; (d) *i*=4.

By adjusting some parameters of emitters, such as pulse width and output power, signals that are different from *y*_*i*_(*i*=1,2, ⋯, 500) can be obtained. Suppose that sample *Ix*_*i*_(*i*=1,2,3,4) is randomly selected from these signals for identification and the dimension of *Ix*_*i*_ is 10240. *x*_*kl*_ represents the *l*th training sample in class *k*, *Bx*_*kl*_ is the label matrix. First, $\bar{I}x_{i}=\left[Ix_{i}\left(1\right)\text{,}\;\;Ix_{i}\left(1\right)+10\text{,}\;\;\cdots \text{,}\;\;Ix_{i}\left(1\right)+10230\right]$ can be obtained according to the method of adding time window. Then, the single-sided amplitude spectrum of center *c*_*k*_ for class *k* is shown in Figure 9; *k*=1,2, ⋯, 5. The double-sided amplitude spectrum of $\bar{I}x_{i}\left(i=1,2,3,4\right)$ is shown in Figure 10. Finally, the identity of $\bar{I}x_{i}\left(i=1,2,3,4\right)$ is preliminary determined by observing the similarity between these amplitude spectrum.

It is observed that the amplitude spectrum of $\bar{I}x_{1}$ is similar to that of the signals in class 2 or class 3, and $\bar{I}x_{2}$ is likely to belong to class 5. But, the class of $\bar{I}x_{3}$ and $\bar{I}x_{4}$ is difficult to determine only by observation. At this time, it is necessary to carry out correlation test for these samples. The test results are shown in Table 1 when *N*_2_=300 and *T*=100.

**Figure 11:**
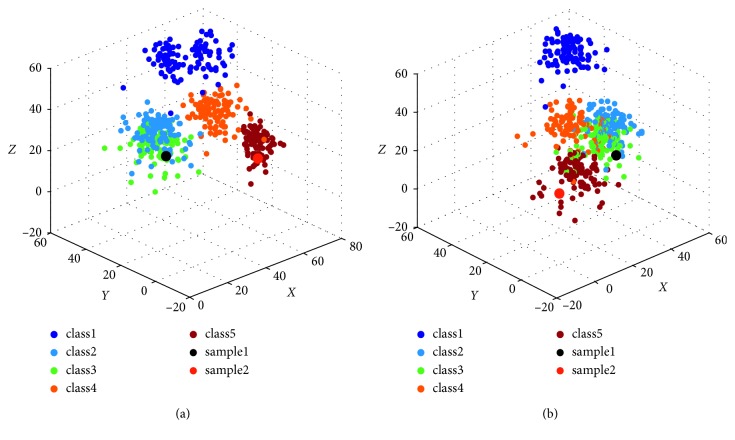
The identified results of [*Ix*_*i*_(*a*_1_)*Ix*_*i*_(*a*_2_)*Ix*_*i*_(*a*_3_)], *i*=1,2. (a) *a*_1_=41, *a*_2_=256, *a*_3_=682  and  *i*=1; (b) *a*_1_=35, *a*_2_=709, *a*_3_=929  and  *i*=2.

Table 1 shows that *r*_12_=0.9704 and *r*_13_=0.9945, which are all greater than 0.95. So, $\bar{I}x_{1}$ might be the element of class 2 or class 3. $\bar{I}x_{2}$ should be classified into class 5. These results are consistent with the observations. $\bar{I}x_{3}$ and $\bar{I}x_{4}$ should be removed before formal classification. Therefore, it is necessary to identify $\bar{I}x_{1}$ and $\bar{I}x_{2}$ by using PNN. *L* points are selected as training samples in each class to train PNN, *L*=70. Finally, The trained PNN is used to judge the identity of $\bar{I}x_{1}$ and $\bar{I}x_{2}$. Since *Ix*_1_ corresponds to $\bar{I}x_{1}$, *Ix*_2_ corresponds to $\bar{I}x_{2}$, we can obtained the identity of *Ix*_1_ and *Ix*_2_ by identifying $\bar{I}x_{1}$ and $\bar{I}x_{2}$. The identified results are shown in Figure 11 when *σ*=1.

In order to obtain intuitive results, the data on columns 41, 256, 682 of *Ix*_1_ are selected to form a three-dimensional array [*Ix*_1_(41)  *Ix*_1_(256)  *Ix*_1_(682)]. Data on the same columns of *Ac* are selected to form a matrix of size 500 × 3. All data are drawn in Figure 11(a). Obviously, *Ix*_1_ should be the element in class 3 (black solid ball) and *Ix*_2_ should be the element in class 5 (red solid ball). The same results can also be obtained when the data on columns 35, 709, and 929 of *Ix*_1_ and *Ac* are selected. The corresponding results are drawn in Figure 11(b).

## 5. Comparative Experiments

**Figure 12:**
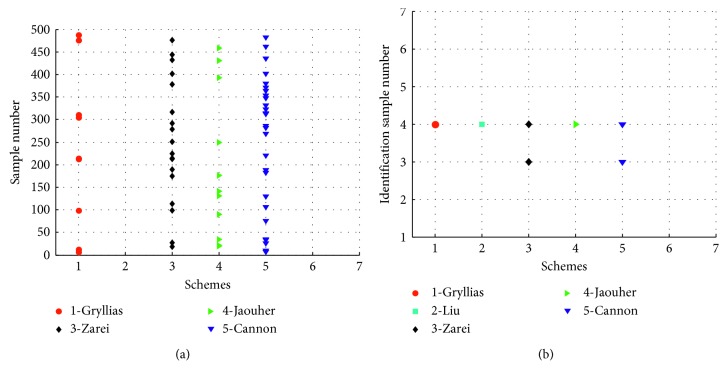
The samples that cannot be judged correctly by different schemes. (a) The samples that cannot be correctly classified. (b) The samples that cannot be correctly identified.

The performance of the proposed algorithm can be reflected through comparative experiments. Therefore, it is necessary to compare it with some usual identification methods, such as support vector machine based schemes [32], particle swarm optimization and support vector machine based schemes [12], artificial neural networks and intelligent filter based schemes [33], PNN and simplified fuzzy adaptive resonance theory map neural networks based schemes [15], and fuzzy c-means based schemes [34]. When the class label of each signal is known, the performance of the above methods can be compared by calculating the classification accuracy and the identification accuracy. The comparison results are shown in Table 2. The samples that cannot be correctly judged are shown in Figure 12.

Table 2 shows that the classification accuracy obtained from Liu's and our methods is 100%. The value of identification accuracy obtained from the proposed method also reaches 100%.  Figure 12(a) shows that some algorithms fail to give the correct classification results for some signals. In terms of signal classification, the number of samples without correct classification for each method is 9, 0, 18, 11, 27, and 0. By comparison, the identification accuracy derived from Zarei's and Cannon's scheme is poor, and they fail to identify the sample 3 and 4 (Figure 12(b)). This is mainly because artificial neural networks and fuzzy c-means algorithm can give a judgment result for each signal, whether or not the signal belongs to the determined classes. Although the PNN has some similar problems, they can be skillfully solved by the method of Bivariable correlation analysis. It makes the proposed method have unique advantages in signal identification.

## 6. Concluding Remarks

It is an indisputable fact that the probabilistic neural networks can be used to classify and identify patterns. It has a wide range of application, including the identification of emitter signals. In this paper, a novel classification and identification scheme, which is designed by the WCM and the PNN with correlation analysis, has been proposed for emitter signals. The scheme starts with self-adaptive filtering processing and spectrum analysis, then the WCM and clustering validity indexes including CH, Silh, DB, and Gap are utilized to determine the range of the optimal number of clusters. For different classification number *K*, 70 samples nearby each center are selected as training samples to establish PNN classifiers. Finally, the optimal PNN classifier which is used to identify signals is determined by the maximum of the classification validity index *D*(*K*). At this stage, the method of Bivariable correlation analysis is cleverly used to improve the identification accuracy of PNN classifier. Experiments show that the proposed method can obtain higher accuracy, and it is more stable than other schemes in identification problems.

Finally, it should be pointed out that the scheme presented above is mainly used to classify the obtained signals, which are derived from some pulse emitters. The classification and identification of signals derived from some continuous wave emitters or the mixed emitters of these two types are the next topic to be studied. In addition, the proposed method can also be used to identify other signals, such as, biomedical signals and monitoring signals of digital virtual assets. When the data set to be classified are not numeric, they can first be converted to binary string and then converted to the required format.

## Figures and Tables

**Algorithm 1:**
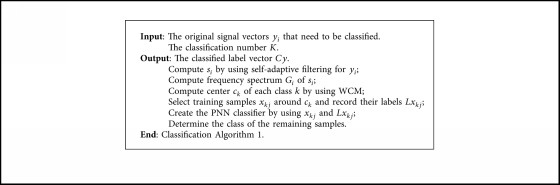
Classification Algorithm 1.

**Algorithm 2:**
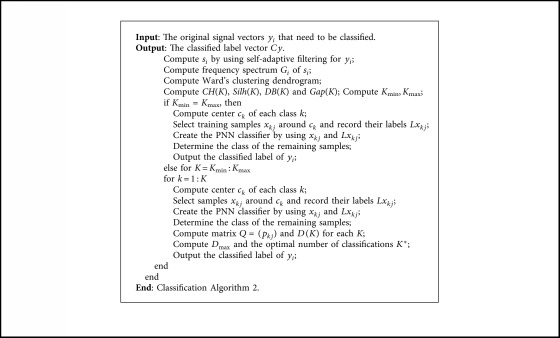
Classification Algorithm 2.

**Algorithm 3:**
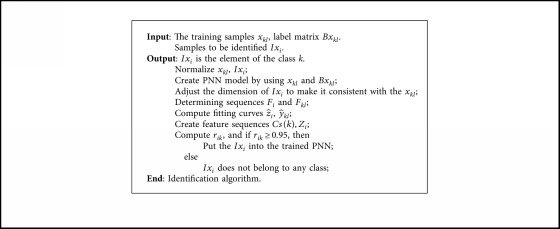
Identification algorithm.

**Table 1:** Correlation test of the samples to be identified.

*r*_*ik*_	Class 1	Class 2	Class 3	Class 4	Class 5
$\bar{I}x_{1}$	0.3687	0.9704	0.9945	0.5473	0.6910
$\bar{I}x_{2}$	0.7991	0.7447	0.6674	0.7882	0.9941
$\bar{I}x_{3}$	0.2364	0.8907	0.7982	0.5202	0.5583
$\bar{I}x_{4}$	0.7864	0.3572	0.3083	0.6887	0.6841

**Table 2:** Accuracy comparison of different methods (*K* = 5).

Literature	Gryllias's [32]	Liu's [12]	Zarei's [33]	Jaouher's [15]	Cannon's [34]	This work
Classification accuracy (%)	98.20	100.00	96.40	97.80	94.60	100.00
Identification accuracy (%)	75.00	75.00	50.00	75.00	50.00	100.00

## Data Availability

Data involve secrets and need to be kept confidential.
